# Hierarchy of quantum correlations using a linear beam splitter

**DOI:** 10.1038/s41598-018-34463-y

**Published:** 2018-11-02

**Authors:** Haleema Sadia Qureshi, Shakir Ullah, Fazal Ghafoor

**Affiliations:** Department of Physics, COMSATS University, Islamabad, 44000 Pakistan

## Abstract

Beam splitters are important components in numerous tasks of quantum information protocols used either in simple or in an interferometric arrangement or together with other quantum systems. This report shows interesting aspects of the quantum correlations of two-mode Gaussian state (TMGS) for the photons retrieved via a linear beam splitter when they are initially employed at the input of either pure or mixed two single-mode Gaussian states (TSMGSs). The quantum correlations obey the boundaries of quantum non-locality, steering, entanglement and discord for pure input states. Though Bell inequality does not violate, quantum steering, entanglement and discord exist in the quantum state evolved by the beam splitter when input states become mixed. Specifically, the quantum steering, entanglement and discord persist to some degrees against the thermal photon number, the Bell inequality is, nonetheless, obeyed by the quantum state except in a very sharp regime.

## Introduction

After the local-realism theory conjectured by Einstein, Podolsky and Rosen (EPR)^1^, scientists paid much attention to define quantum correlations, e.g. Bell non-locality, quantum steering, quantum entanglement, quantum discord, and quantum coherence^2–16^, mainly due to their useful applications to quantum information processing. In general, all kinds of quantum correlations satisfy the conditions: quantum coherence ⊇ quantum discord ⊇ quantum entanglement ⊇ quantum steering ⊇ Bell non-locality^3,17–24^ where, for example, *P* ⊇ *Q* means *P* is the superset of *Q*. This reveals that steerable states are a strict subset of the entangled states and a strict superset of the states that may exhibit Bell non-locality and similarly to the others, the so-called hierarchy relations of the quantum correlations.

Quantum entanglement between states of a composite system emerged as an interesting resource for the advancement of both basic physics and applications. Today, entanglement has proved to be the building blocks in devising various techniques in the science of quantum information processing^25–27^. The phenomenon of quantum steering investigated by Schrodinger^28^ in the subsystems of a composite quantum system, occupies an intermediate position between Bell non-locality and entanglement in modern quantum information theory. Mathematical inequalities for quantum steering measurement have been proposed for continuous- and discrete- variable quantum systems^4,7,19–21,29–31^. On the other hand, Bell’s^32^ local realist model for measurement of EPR state was originally formulated as a function in the Wigner representation using measurements of the joint classical-probability. However, the Wigner function becomes quantum correlated when the quantum system is subjected to parity measurements. On the basis of displacement operation and parity measurements^33^, Bell non-locality in the field state of two spatially separated cavities has been studied^34^. Using Schmidt form for the entangled non-orthogonal states^35^ and quadrature-phase states^36^, Bell non-locality has been reported. Temporal evolution of non-local two-mode squeezed state is investigated in a thermal environment as well^37,38^. The Bell and Bell type inequalities in the quantum state of various systems has already passed all addressed loop-hole^3,39^. Furthermore, as compared to quantum entanglement, disentangled quantum systems exhibit another kind of quantum correlations, known as quantum discord^13,14,17^, is useful in quantum information theory. Beyond the quantum entanglement phenomenon, quantum discord quantifies quantumness of the correlations associated with the quantum systems^40,41^. Such measurements provide us with the amount of mutual information that is not locally accessible of multipartite systems. Quantum discord has been extensively investigated in several physical systems such as spin chains^42–44^, cavity quantum electrodynamics^45–47^ and quantum dots^48^.

Continuous variable states, as compared to discrete variable, are easy to manipulate experimentally^25^. The Gaussian type states which are one of the important class of the continuous variable states, usable at the input of a linear beam splitter, have been realized using techniques of optical parametric converter^49^, parametric down converter^50^ and two-mode squeezing effect^25^. The approach of quantifying the quantum correlations of continuous variable^7,25,40,41,51^ and discrete variable^3,5,11,13^ states have been studied. In the present study, we investigate in detail the quantum correlations of continuous variable states through a beam splitter. Linear beam splitter is the basic component in various tasks of quantum information protocols, such as quantum entanglement^52–54^, teleportation^55^, steering^21^, used either in simple or in an interferometric arrangement or together with other types of quantum systems. The study of quantum correlations of continuous variable states with respect to a linear beam splitter in simple is interesting from perspective of basic physics and applications.

**Figure 1:**
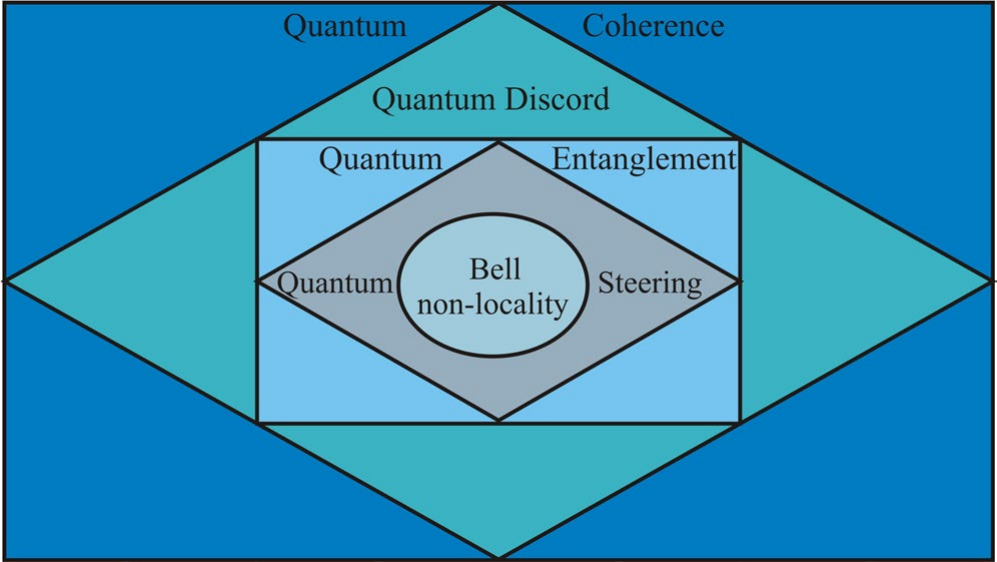
Quantum correlations: quantum coherence ⊇ quantum discord ⊇ quantum entanglement ⊇ quantum steering ⊇ Bell non-locality, where *P* ⊇ *Q* represent *P* is the superset of *Q* etc.

In this report, we study important aspects of the quantum correlations of photons retrieved via a linear beam splitter employing either pure or mixed TSMGSs of continuous variable system initially at the input. Boundaries of the quantum correlations of photons at the output obey quantum non-locality, steering, entanglement and discord in presence of the pure input states. On contrary, the Bell inequality does not violate, however, the quantum steering, entanglement and discord exist in some regime of angle *θ* of the beam splitter when the input states are mixed. Against the thermal photon number, the quantum steering, entanglement and discord persist to some degrees whereas the Bell inequality is obeyed except in a very sharp regime^40,41^. In this case, we find that all the four kinds of the quantum correlations form a hierarchy (see Fig. 1).

## Results

### Two single-mode Gaussian states at inputs

We employ TSMGSs of photons at the input of a lossless beam splitter with complex probability amplitudes (*δ*_1_, *δ*_2_). The characteristic function for the two separable states is written as

1 $$\chi ({\delta }_{1},{\delta }_{2})=\exp (-\frac{1}{2}{\delta }^{\dagger }{V}_{in}\delta ),$$

where ${\delta }^{\dagger }=({\delta }_{1}^{\ast },{\delta }_{1},{\delta }_{2}^{\ast },{\delta }_{2})$ is the row matrix of complex amplitudes, and $${V}_{in}=(\begin{array}{cc}A & 0\\ 0 & B\end{array})$$ is the covariance matrix for TSMGSs. In last matrix, $$A=(\begin{array}{cc}{h}_{1} & {h}_{11}\\ {h}_{11}^{\ast } & {h}_{1}\end{array})$$ and $$B=(\begin{array}{cc}{h}_{2} & {h}_{22}\\ {h}_{22}^{\ast } & {h}_{2}\end{array})$$ represent the sub covariance matrices associated with the first and second input beams, respectively, where the elements *h*_*i*_ are real and ${h}_{ii}=|{h}_{ii}|{e}^{i{\varphi }_{i}}$ are complex (*i* = 1, 2). Degree of non-classicality^56^ *τ* for continuous variables Gaussian state is defined as ${\tau }_{i}=\,{\rm{\max }}\{\mathrm{0,}\frac{1}{2}-{\lambda }_{i}\}$, where ${\lambda }_{i}={h}_{i}-|{h}_{ii}|$ is the minimum eigenvalue of sub-matrices *A* and *B*. The range of non-classicality *τ* satisfies the limit $0\le \tau \le \mathrm{1/2}$. Purity *μ* for continuous variables Gaussian state is however, defined in terms of density matrix *ρ* as $\mu (\rho )=Tr[{\rho }^{2}]$ with *μ* being in the regime $0\le \mu \le 1$. Since quantum state with *μ* < 1 is mixed and with *μ* = 1 pure, the purity^57^ of the input states becomes as ${\mu }_{i}=\frac{1}{2\sqrt{{\rm{\det }}\,{v}_{i}}}$, where *v*_*i*_ is the covariance matrix of subsystems *A* and *B*. Hence, the elements of the input covariance matrix are written in terms of non-classicality *τ*_*i*_ and purity *μ*_*i*_ as

2 $$\begin{array}{rcl}{h}_{i} & = & \frac{1}{4{\mu }_{i}^{2}\mathrm{(1}-2{\tau }_{i})}+\frac{1}{4}\mathrm{(1}-2{\tau }_{i}),\\ |{h}_{ii}| & = & \frac{1}{4{\mu }_{i}^{2}\mathrm{(1}-2{\tau }_{i})}-\frac{1}{4}\mathrm{(1}-2{\tau }_{i}\mathrm{).}\end{array}$$

At the output, characteristic function for the TMGS with complex amplitudes *ξ*_1_ and *ξ*_2_ becomes as

3 $$\chi ({\xi }_{1},{\xi }_{2})=\exp (-\,\frac{1}{2}{\xi }^{\dagger }{V}_{AB}\xi )\mathrm{.}$$

where $${\xi }^{\dagger }=({\xi }_{1}^{\ast },{\xi }_{1},{\xi }_{2}^{\ast },{\xi }_{2})$$ and

4 $${V}_{AB}={U}_{BS}^{\dagger }{V}_{in}{U}_{BS}=(\begin{array}{cccc}{f}_{1} & {f}_{11}^{\ast } & {f}_{12}^{\ast } & {f}_{3}^{\ast }\\ {f}_{11} & {f}_{1} & {f}_{3} & {f}_{12}\\ {f}_{12} & {f}_{3}^{\ast } & {f}_{2} & {f}_{22}^{\ast }\\ {f}_{3} & {f}_{12}^{\ast } & {f}_{22} & {f}_{2}\end{array}),$$

is the two-mode output covariance matrix. In Eq. (4) *U*_*BS*_ is the 4 × 4 unitary transformation matrix of the beam splitter given by

5 $${U}_{BS}=(\begin{array}{cccc}\cos \,\theta  & 0 & -\sin \,\theta {e}^{i\phi } & 0\\ 0 & \cos \,\theta  & 0 & -\sin \,\theta {e}^{-i\phi }\\ \sin \,\theta {e}^{-i\phi } & 0 & \cos \,\theta  & 0\\ 0 & \sin \,\theta {e}^{i\phi } & 0 & \cos \,\theta \end{array}),$$

where *θ* represents the orientation of the beam splitter, *φ* is the phase difference between reflected and transmitted beams, and ${f}_{1}={h}_{1}\,{\cos }^{2}\theta +{h}_{2}\,{\sin }^{2}\theta$, ${f}_{11}={h}_{11}\,{\cos }^{2}\theta +{h}_{22}\,{\sin }^{2}\theta {e}^{-2i\phi }$, ${f}_{2}={h}_{1}\,{\sin }^{2}\theta +{h}_{2}\,{\cos }^{2}\theta$, ${f}_{22}={h}_{11}\,{\sin }^{2}\theta {e}^{2i\phi }+$ ${h}_{22}\,{\cos }^{2}\theta$, ${f}_{3}=({h}_{11}{e}^{i\phi }-{h}_{22}{e}^{-i\phi })\sin \,\theta \,\cos \,\theta$, and ${f}_{12}=({h}_{1}^{\ast }-{h}_{2}^{\ast })\sin \,\theta \,\cos \,\theta {e}^{-i\phi }$. Below, we exploit Bell non-locality, quantum steering, entanglement and Gaussian discord of the photons retrieved through the beam splitter using TSMGSs of various properties initially at the input. The Bell’s combination^33,37^ for the TMGS at the output of the beam splitter is evaluated as

6 $$ {\mathcal B} =P\{1+2\exp [-{b}_{2}{R}_{1}]\mathrm{ln}(z)-\exp [-\,\frac{2\,\mathrm{ln}(z)}{{R}_{1}{R}_{2}}]\}.$$

All the terms on the right side of Eq. (6) are defined in Eq. (19), as presented in Methods. The Bell’s combination ${\mathcal B}$ obeys the range of the inequality $-2 <  {\mathcal B}  < 2$ when the quantum state becomes separable. Otherwise, it is inseparable.

Quantum steering^7,19^ owns the place between entanglement and Bell non-locality in the quantum correlations. How much *A* steers *B* for the covariance matrix *V*_*AB*_ of the quantum state is quantified by

7 $${S}^{A\to B}=\frac{1}{2}{\mathrm{log}}_{2}[\frac{{M}_{1}{\cos }^{4}\theta +{M}_{2}{\sin }^{4}\theta +{M}_{3}{\cos }^{2}\theta \,{\sin }^{2}\theta }{4{M}_{1}{M}_{2}}],$$

where *A*(*B*) being the subsystem for measurement of the first (second) party, ${M}_{1}={h}_{1}^{2}-{|{h}_{11}|}^{2}$, ${M}_{2}={h}_{2}^{2}-{|{h}_{22}|}^{2}$ and ${M}_{3}=2{h}_{1}{h}_{2}-{h}_{11}^{\ast }{h}_{22}{e}^{-2i\phi }-{h}_{11}{h}_{22}^{\ast }{e}^{2i\phi }$. The degree of entanglement for inseparable states is characterized by logarithmic negativity^11,51^ which for the TMGS appears as

8 $${E}_{n}=-\,{\mathrm{log}}_{2}[\sqrt{M-\sqrt{{M}^{2}-16{M}_{1}{M}_{2}}}],$$

where $$M=({M}_{1}+{M}_{2})\,\mathrm{(1}+\,\cos \,4\theta )+{M}_{3}\mathrm{(1}-\,\cos \,4\theta )$$.

The Gaussian quantum discord^40,41^, the superset of all the above mentioned quantum correlations, is measured as

9 $$D=F(\sqrt{\beta })-F({\lambda }_{-})-F({\lambda }_{+})+F(\sqrt{\varepsilon }),$$

using the set of positive operator valued measurements on the subsystem *B*, where $F(x)=\frac{x+1}{2}\,{\mathrm{log}}_{2}(\frac{x+1}{2})-$ $\frac{x-1}{2}{\mathrm{log}}_{2}(\frac{x-1}{2}),$

10 $$\varepsilon =\{\begin{array}{ll}\frac{2{\gamma }^{2}+(\beta -\mathrm{1)(}\delta -\alpha )+2|\gamma |\sqrt{{\gamma }^{2}+(\beta -\mathrm{1)(}\delta -\alpha )}}{{(\beta -\mathrm{1)}}^{2}} & {\rm{if}}\,{(\delta -\alpha \beta )}^{2}-(\beta +\mathrm{1)}\,(\alpha +\delta )\le 0\\ \frac{\alpha \beta -{\gamma }^{2}+\delta -\sqrt{{\gamma }^{4}+{(\delta -\alpha \beta )}^{2}-2{\gamma }^{2}(\delta +\alpha \beta )}}{2\beta } & {\rm{otherwise}}\end{array}\},$$

$\alpha ={\rm{\det }}\,{\mathbb{A}}$, $\beta ={\rm{\det }}\,{\mathbb{B}},$ $\gamma ={\rm{\det }}\,{\mathbb{C}},$ $\delta =4{\rm{\det }}{V}_{AB},$ and *λ*_±_ being the symplectic eigenvalues of the two-mode covariance matrix *V*_*AB*_, given by $2{\lambda }_{\pm }^{2}={\rm{\Delta }}\pm \sqrt{{{\rm{\Delta }}}^{2}-4{\rm{\det }}{V}_{AB}}$ with ${\rm{\Delta }}={\rm{\det }}\,{\mathbb{A}}+{\rm{\det }}\,{\mathbb{B}}+2{\rm{\det }}\,{\mathbb{C}}$. To obtain the smaller symplectic eigen value ${\tilde{\lambda }}_{-}$ under the partially transposed state, we replace *γ* with −*γ* in the expression of *λ*_−_. Towards this measurement, TMGS becomes inseparable if the condition *D* > 1, is satisfied. The quantum state is either separable or inseparable when the measurement obeys the condition 0 ≤ *D* ≤ 1^40,41^.

**Figure 2:**
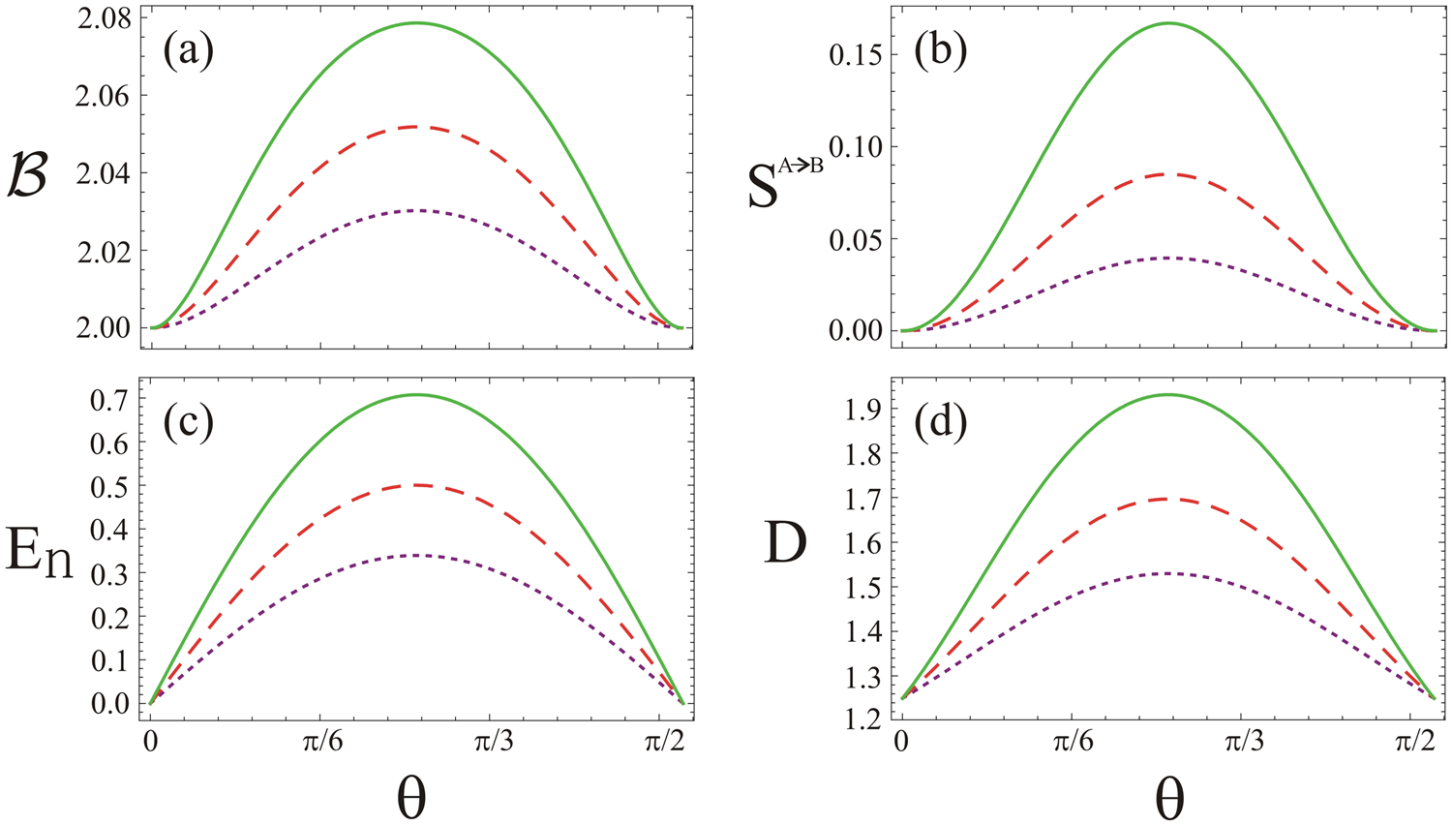
The quantum correlations of TMGS at the output of beam splitter for fixed values of *μ*_1_ = *μ*_2_ = 1, *τ*_1_ = 0.1, *ϕ*_1_ = −*π* and ${\varphi }_{2}=\phi =\pi$. (**a**) Bell’s combination, (**b**) quantum steering, (**c**) quantum entanglement, and (**d**) quantum discord against beam splitter angle *θ*. In all the plots the dotted-purple, dashed-red and solid-green lines are for ${\tau }_{2}=0.25$, 0.3 and 0.35, respectively.

As shown in Fig. 2, response of the quantum non-locality, steering, entanglement and quantum Gaussian discord are plotted against the beam splitter angle *θ* using *μ*_1_ = *μ*_2_ = 1, *τ*_1_ = 0.1, *ϕ*_1_ = −*π* and *ϕ*_2_ = *φ* = *π* for various values of the non-classicality *τ*_2_. Apparently, influence of all the four kinds of the quantum correlations is maximal for the choice of $\theta =\frac{\pi }{4}$. However, the influence minimizes gradually when the angle *θ* deviates from $\frac{\pi }{4}$. Moreover, effect of the correlations grows with increasing non-classicality *τ*_2_ except in the regimes *θ* = 0 and $\frac{\pi }{2}$. In Fig. 2(a), Bell’s combination of the evolved quantum state violates the inequality $-2 <  {\mathcal B}  < 2$ which predicts the quantum system is non-local. The subsystem *A*, on the other side, steers *B* showing the quantum steering *S*^*A*→*B*^ effect in the system (see Fig. 2(b)). Like Bell non-locality and quantum steering, entanglement between the two subsystems exhibits as well (see Fig. 2(c)). To get insight beyond the entanglement phenomenon, the Gaussian quantum discord *D* is next investigated as shown in Fig. 2(d). Obviously, the condition *D* > 1 is obeyed, leads to inseparability of the TMGS. This means that maximal information can be extracted from the quantum system. Finally, we conclude that the four kinds of the quantum correlations reveal similar characteristics in their own respects i.e. the entangled pure states are steerable and also violate Bell inequality whereas the Gaussian quantum discord shows enhanced profile for the quantum correlations produced by the beam splitter.

**Figure 3:**
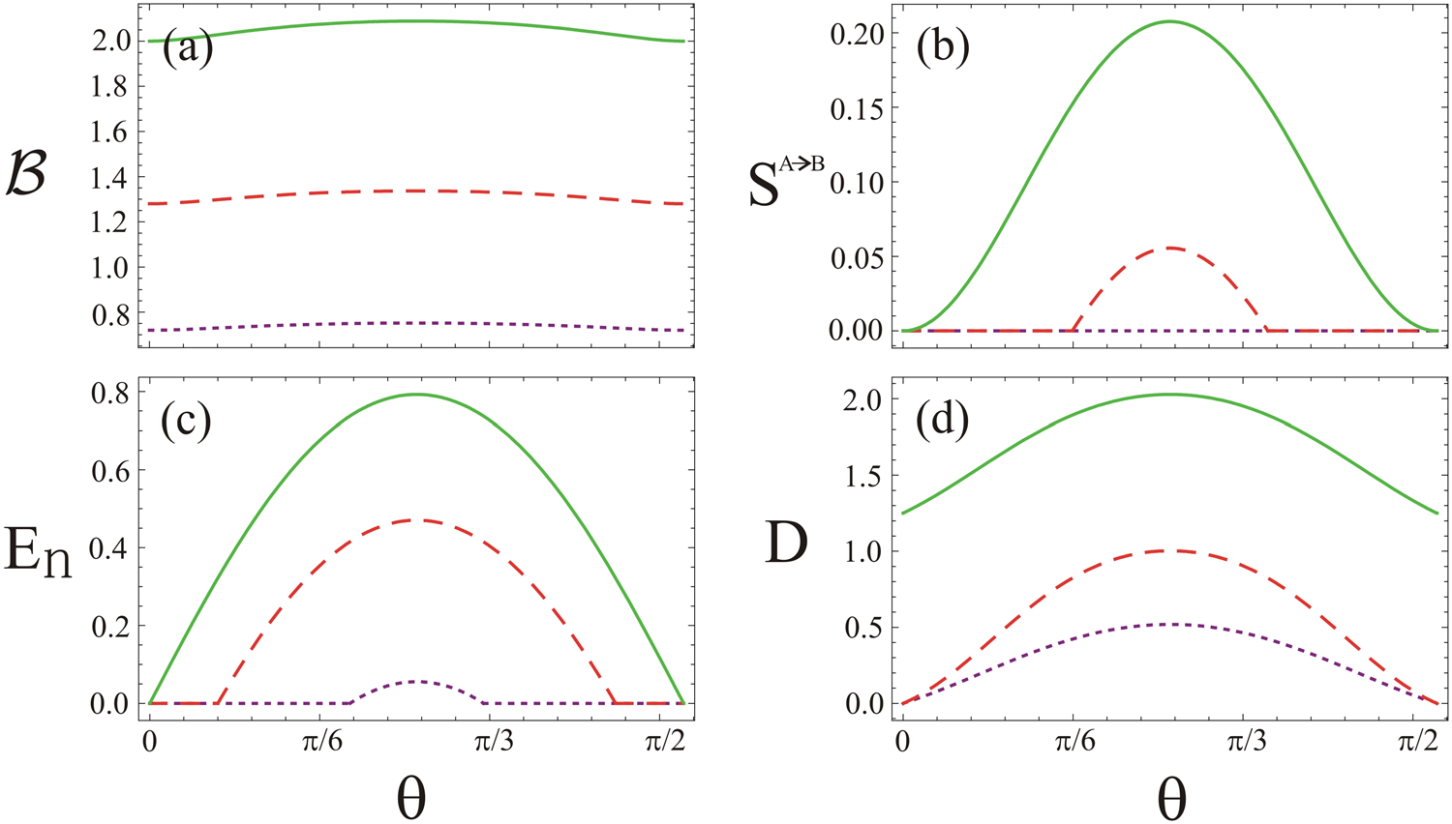
The quantum correlations of TMGS at the output of beam splitter for fixed values of *τ*_1_ = 0.2, *τ*_2 _= 0.4 *ϕ*_1_ = −*π* and *ϕ*_2_ = *φ* = *π*. (**a**) Bell’s combination, (**b**) quantum steering, (**c**) quantum entanglement, and (**d**) quantum discord against beam splitter angle *θ*. In all the plots the dotted-purple, dashed-red and solid-green lines are for *μ*_1_ = *μ*_2_ = 0.6, 0.8 and 1, respectively.

On the other hand, Bell inequality does not violate when the input states of the beam splitter are chosen mixed states (see Fig. 3(a)). This aspect of the study is in accord with the ones reported in ref.^58^. We further show the quantum steering *S*^*A*→*B*^ in Fig. 3(b) using the identical conditions of the input states as used in Fig. 3(a). In this case, the quantum state is non steerable when the purities of the input states become as *μ*_1_ = *μ*_2_ = 0.6. However, for *μ*_1_ = *μ*_2_ = 0.8, the quantum steering is zero before the angle $\theta =\frac{\pi }{6}$ of the beam splitter and suddenly exhibits in the system beyond this angle. After maximal effect is reached, the steering diminishes gradually and vanish at $\frac{\pi }{3}$. In comparison to Bell non-locality and quantum steering, we next show entanglement between the TMGS using logarithmic negativity *E*_*n*_ (see Fig. 3(c)). In this case, entanglement exists in different ranges of *θ* with distinct purities, for instance when *μ*_1_ = *μ*_2_ = 0.6, *E*_*n*_ lies in a short range $\frac{2\pi }{9}\le \theta \le \frac{5\pi }{18}$. This result reveals that the quantum state of the system is non steerable (Fig. 3(b)) and Bell inequality does not violate as well (Fig. 3(a))^19,20^. Further, the quantum discord *D* satisfies the condition 0 ≤ *D* ≤ 1 in the entire range of *θ* as shown in Fig. 3(d). Here, in a region, where the logarithmic negativity is zero, the Gaussian quantum discord appears non zero^24^. The behavior of the discord does not tell us whether the state is separable or inseparable. Looking at the entanglement however shows that in this case the state is separable when *E*_*n*_ = 0 otherwise it is inseparable (see Fig. 3(c)). However, the quantum discord in the system always shows maximal inseparability in the photons when the input photons employed are in pure states. Since maximal quantum discord *D* means maximal quantum entanglement, maximal quantum information from the system may be extracted in this case.

To conclude with this discussion, we find that when there is entanglement in the system, it must always exhibits quantum discord. Contrarily, the system exhibiting quantum discord does not necessarily show the entanglement phenomenon. Likewise, every violation of Bell inequality shows quantum steering in the system and quantum steering is a witness of entanglement. In both the cases, the system showing the latter one may not reveal the former. Hence, in presence of mixed input states, the boundaries of all the four kinds of the quantum correlation form a hierarchy.

### An arbitrary single-mode Gaussian and a thermal state at inputs

Now as an example, we consider the first input an arbitrary single-mode Gaussian state and the second input a thermal state, the covariance matrix for the two states is ${V}_{in}=A\oplus C,$ with sub matrices $$A=(\begin{array}{cc}{h}_{1} & {h}_{11}\\ {h}_{11}^{\ast } & {h}_{1}\end{array})$$ and $$C=(\begin{array}{cc}n+\mathrm{1/2} & 0\\ 0 & n+\mathrm{1/2}\end{array})$$. After the action of beam splitter, the evolved state with covariance matrix becomes

11 $${V}_{AC}=(\begin{array}{cccc}{k}_{1} & {k}_{11}^{\ast } & {k}_{12}^{\ast } & {k}_{3}^{\ast }\\ {k}_{11} & {k}_{1} & {k}_{3} & {k}_{12}\\ {k}_{12} & {k}_{3}^{\ast } & {k}_{2} & {k}_{22}^{\ast }\\ {k}_{3} & {k}_{12}^{\ast } & {k}_{22} & {k}_{2}\end{array}),$$

where ${k}_{1}={h}_{1}\,{\cos }^{2}\theta +(n+\frac{1}{2}){\sin }^{2}\theta ,$ ${k}_{11}={h}_{11}^{\ast }\,{\cos }^{2}\theta ,$ ${k}_{2}={h}_{1}\,{\sin }^{2}\theta +(n+\frac{1}{2}){\cos }^{2}\theta ,$ ${k}_{22}={h}_{11}^{\ast }\,{\sin }^{2}\theta {e}^{2i\phi },$ ${k}_{3}={h}_{11}^{\ast }\,\sin \,\theta \,\cos \,\theta {e}^{i\phi },$ and ${k}_{12}=({h}_{1}-n-\frac{1}{2})\sin \,\theta \,\cos \,\theta {e}^{-i\phi }\mathrm{.}$ Using Eqs. (6–9), it is easy to estimate expressions for Bell inequality, quantum steering, quantum entanglement and quantum discord explicitly.

**Figure 4:**
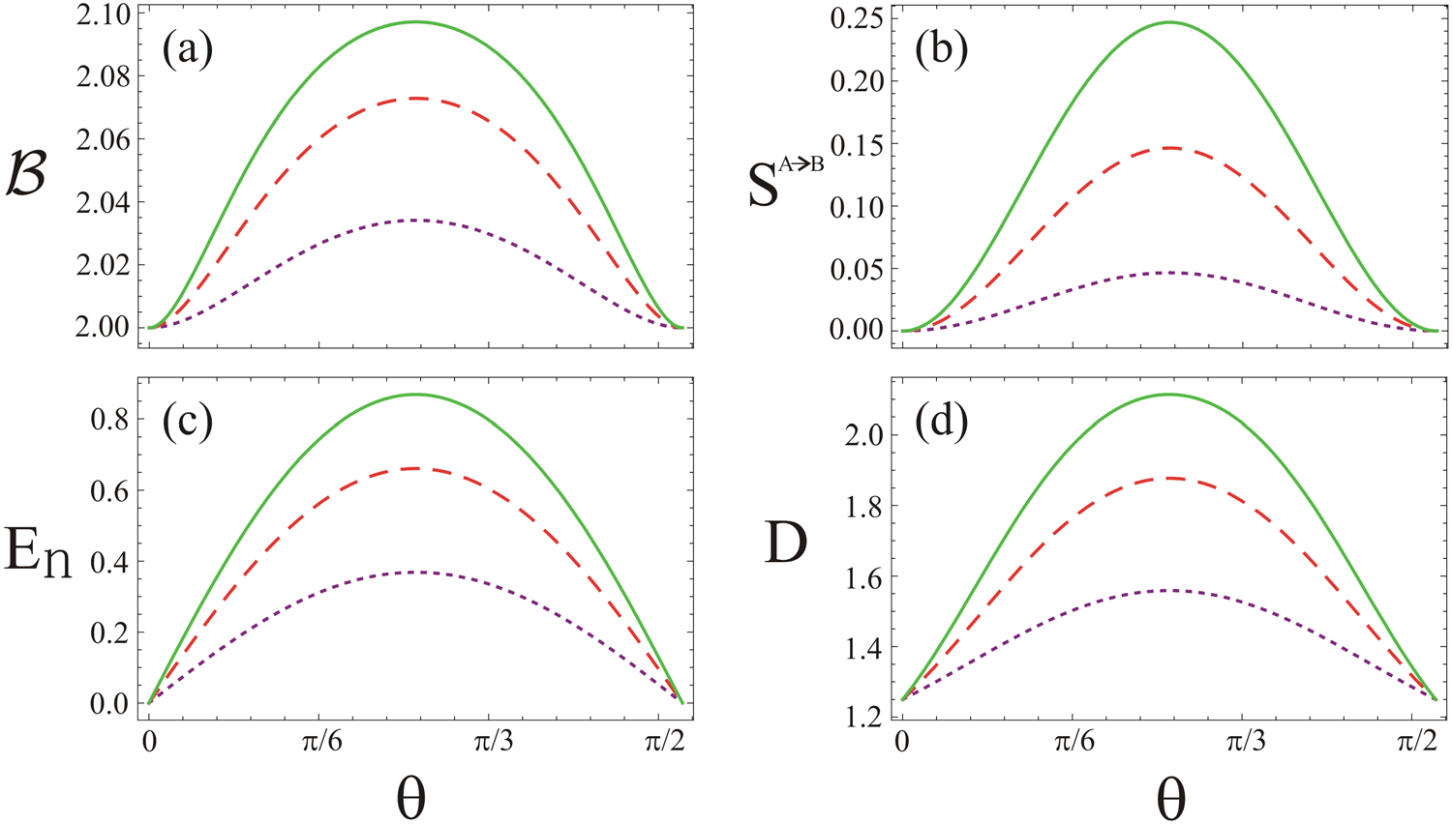
The quantum correlations of TMGS at the output of beam splitter for fixed values of *n* = 0, *μ* = 1, *ϕ* = 0 and *φ* = *π*. (**a**) Bell’s combination, (**b**) quantum steering, (**c**) quantum entanglement, and (**d**) quantum discord against beam splitter angle *θ*. In all the plots the dotted-purple, dashed-red and solid-green lines are for *τ* = 0.2, 0.3 and 0.35, respectively.

**Figure 5:**
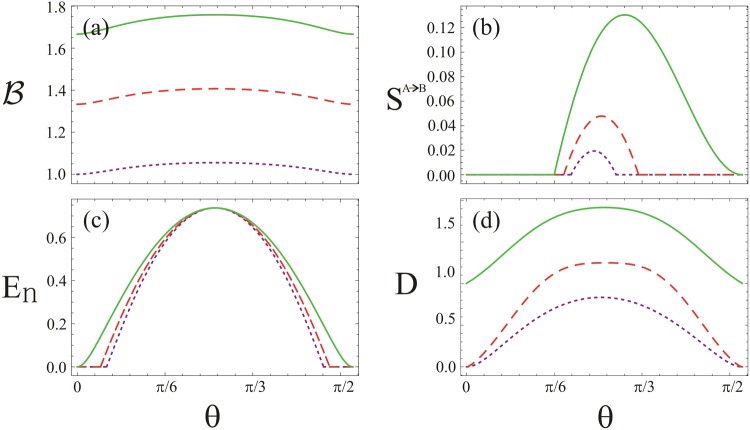
The quantum correlations of TMGS at the output of beam splitter for fixed values of *n* = 0.1, *τ* = 0.35, *ϕ* = 0 and *φ* = *π*. (**a**) Bell’s combination, (**b**) quantum steering, (**c**) quantum entanglement, and (**d**) quantum discord against beam splitter angle *θ*. In all the plots the dotted-purple, dashed-red and solid-green lines are for *μ* = 0.6, 0.8 and 1, respectively.

Response of the four kinds of the quantum correlations against the angle *θ* is further shown in Fig. 4 for *n* = 0, *μ* = 1, *ϕ* = 0, and *φ* = *π* along with various non-clasicality *τ* of the input states. As shown in Fig. 4(a), the Bell inequality violates except at the angle *θ* = 0 and $\frac{\pi }{2}$ of the beam splitter. The influence of the four kinds of the quantum correlations indicate enhanced profile with the non-classicality *τ*. In each case of the correlations, the behavior is qualitatively similar to the ones reported in Fig. 2. In comparison to the study presented in Fig. 3, we further show Bell inequality, quantum steering, entanglement and discord against the angle *θ* of the beam splitter in the presence of thermal photons using *n* = 0.1, *τ* = 0.35 *ϕ* = 0, and *φ* = *π* with various values of the purity *μ* of the input states (see Fig. 5). In this case, effect of the non-local correlations in the system is degraded. As a result, Bell inequality does not violate, examining a local behavior (see Fig. 5(a))^37,38^. The Gaussian state, on the other hand, is steerable (see Fig. 5(b)) under the same parametrization as used in Fig. 5(a). We note that in comparison to the study presented in Fig. 3(b), the quantum steering in the system represents various asymmetrical profile with respect to the maximal steering effect in the system. In Fig. 5(c), generation of entanglement between TMGS occurs in a broader range of the angle *θ* unlike the entanglement behavior generated by the beam splitter as shown in Fig. 3(c). In this case, maxima produced by the beam splitter is independent of the purity of the input state. In both the cases, profile of the entanglement reveals qualitatively similar behavior. Furthermore, quantumness of the TMGS as measured by the Gaussian quantum discord (see Fig. 5(d)) indicates similar characteristics in comparison to the one reported in Fig. 3(d). It is obvious from Fig. 5, the Gaussian quantum discord, quantum entanglement, quantum steering and Bell non-locality represent hierarchy of the quantum correlations as studied in this system.

**Figure 6:**
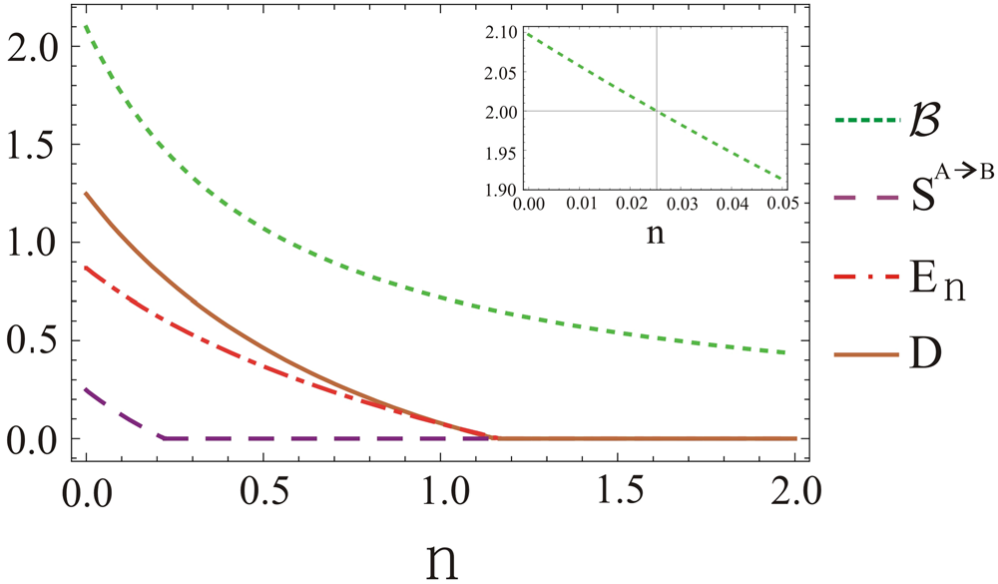
The quantum correlations of TMGS at the output of beam splitter for fixed values of *μ* = 1, *τ* = 0.35, *θ* = *π*/4, *ϕ* = 0 and *φ* = *π*. The dotted-green, dashed-purple, dot-dashed-red and solid-brown lines represent respectively the Bell non-locality, quantum steering, quantum entanglement, and quantum discord against thermal photon number *n*.

Finally we summarize our study in Fig. 6 for the four kinds of the quantum correlations versus the thermal photon number *n* using the other parameters as *μ* = 1, *τ* = 0.35, *ϕ* = 0, and *φ* = *π*. We note that characteristics of the four kinds of the quantum correlations show inverse relation in presence of the thermal photons *n*. In each case, the effect of non-classical correlations decreases as the thermal photons *n* increases at the input of the beam splitter. Bell inequality violates strictly in the smallest regime 0 ≤ *n* < 0.025 and is obeyed when *n* ≥ 0.025 which shows almost total loss of the quantum non-locality in the retrieved TMGS^37,38^. Note that effect of the quantum steering *S*^*A→B*^ in the quantum state vanishes when *n* ≥ 0.22. This means that subsystem *A* steers *B* in the limit 0 ≤ *n* < 0.22. In comparison to ${\mathcal B}$ and *S*^*A*→*B*^, the generation of entanglement between the two modes as measured by the logarithmic negativity *E*_*n*_ lies in the range 0 ≤ *n* ≤ 1.17. In the same limit 0 ≤ *n* ≤ 1.17, the Gaussian quantum discord *D* in the TMGS exhibits an enhanced profile which represents an important indicator to examine the non-classical correlations. Apparently, the results of all the quantum correlations, as displayed on Fig. 6, demonstrate transparently that $D > {E}_{n} > {S}^{A\to B} >  {\mathcal B}$ which means that ${\mathcal B}$ is the subset of *S*^*A*→*B*^ and similarly the others.

## Summary

In this report, we have studied four kinds of the quantum correlations such as Bell non-locality, quantum steering, entanglement and discord via a linear beam splitter using TSMGSs initially at the input. Influence of each one correlation with respect to the others have been analyzed both for pure and mixed input states.

The TMGS evaluated by the beam splitter exhibits non-locality, quantum steering, entanglement and discord, when pure input states are taken into account. The quantum correlation shows similar behavior in each case. Upon increasing the non-classicality of the input modes, the quantum correlations indicate enhanced profile.

On the other hand, the Bell inequality is obeyed by the quantum state when mixed states are employed at the input. Similarly, the quantum state is non steerable when mixedness of the input states are enhanced (see Fig. 3(b)), otherwise, the quantum state is steerable in various ranges of the beam splitter angle *θ*. However in such a situation, quantum entanglement (discord) in the TMGS is obtained in different (whole) ranges (range) of the beam splitter angle *θ*. The effect of the quantum steering, entanglement and discord increases quantitatively with increased purity of the input states. To clearly get insight, the effect of violation of Bell inequality, quantum steering, entanglement and discord is specifically shown against thermal photon number. In this case, the Bell inequality is obeyed except in a very sharp regime of the thermal photons. However, the quantum steering, entanglement and discord still exhibits by the TMGS.

Finally for mixed input states, we identified that Bell non-locality leads to steerability in the system and quantum steering is a witness of entanglement but the reverse is not always true. Similarly, entanglement in the system always exhibits quantum discord but the latter may not necessarily shows the former. Hence, based on these analysis, the hierarchy relation quantum discord ⊇ quantum entanglement ⊇ quantum steering ⊇ Bell non-locality is satisfied by the four kinds of the quantum correlation in the system, where *P* ⊇ *Q* means *P* is the superset of *Q*, stating such that steerable states are a strict subset of the entangled states, and a strict superset of the states that may exhibit Bell non-locality.

The present study may prove extremely availing from perspective of basic physics and its application to numerous quantum information protocols.

## Methods

### Gaussian states

An important class of continuous variable states, in the representation of Wigner function is called a Gaussian state if it characterizes a quasiprobability distribution. Here we consider the phase-space quadratures $\hat{X}=({\hat{x}}_{A},{\hat{p}}_{A},{\hat{x}}_{B},{\hat{p}}_{B})$ for a two-mode quantum system which satisfy the canonical commutation relations $[{\hat{X}}_{i},{\hat{X}}_{j}]=i{{\rm{\Omega }}}_{ij}$, with $${\rm{\Omega }}={\oplus }_{k=1}^{2}(\begin{array}{cc}0 & 1\\ -1 & 0\end{array})$$ being the symplectic form. The first and second statistical moments are the relevant quantities which define the Gaussian state with the mean quadratures $\langle \hat{X}\rangle =X$ and with the elements ${[{V}_{AB}]}_{ij}=\frac{1}{2}\langle {\hat{X}}_{i}{\hat{X}}_{j}+{\hat{X}}_{j}{\hat{X}}_{i}\rangle -\langle {\hat{X}}_{i}\rangle \langle {\hat{X}}_{j}\rangle$ of the covariance matrix *V*_*AB*_. Since the correlations in Gaussian states are independent of local unitary operation, the first moment *X* becomes 0.

For a TMGS, the covariance matrix can be written in block form as $${V}_{AB}=(\begin{array}{cc}{\mathbb{A}} & {\mathbb{C}}\\ {{\mathbb{C}}}^{T} & {\mathbb{B}}\end{array})$$, where ${\mathbb{A}}$ and ${\mathbb{B}}$ are the 2 × 2 covariance matrices of subsystems *A* and *B,* and ${\mathbb{C}}$ is the correlation matrix between the two-modes. In order to describe a physical quantum state, every covariance matrix must satisfy the strong form of uncertainty principle on the canonical operators ${V}_{AB}+i({{\rm{\Omega }}}_{A}\oplus {{\rm{\Omega }}}_{B})\ge 0$.

### Quantum correlations

Bell non-locality^32^ is a channel in which non-local correlated random variables can be generated among distant parties after performing local measurements on their subsystems, such that the correlations are not pre-determined at the source. In order to investigate the non-locality of the output state in the phase space, we explicitly calculate its Wigner function^59^. The Wigner function in terms of the output characteristic function for the output TMGS after the action of beam splitter can be defined as

12 $${W}_{TMGS}({\alpha ^{\prime} }_{1},{\alpha ^{\prime} }_{2})=\frac{1}{{\pi }^{4}}\int \,\int \,{d}^{2}{\xi }_{1}{d}^{2}{\xi }_{2}\chi ({\xi }_{1},{\xi }_{2}){\breve{E}}_{1}{\breve{E}}_{2},$$

where ${\breve{E}}_{i}={e}^{{\xi }_{i}^{\ast }{\alpha ^{\prime} }_{i}-{\xi }_{i}{\alpha ^{\prime} }_{j}^{\ast }}$ (*i* = 1, 2). On substitution of Eq. (3) in Eq. (12), the Wigner function of the quantum system of the beam splitter is evaluated as

13 $$\begin{array}{rcl}{W}_{TMGS}({\alpha ^{\prime} }_{1},{\alpha ^{\prime} }_{2}) & = & \frac{{\pi }^{-2}}{\sqrt{({a}_{1}{a}_{2}-{c}_{1}^{2})({b}_{1}{b}_{2}-{c}_{2}^{2})}}\\  &  & \times \,\exp [4X({|{\alpha }_{1}|}^{2}{a}_{2}{\sin }^{2}{\phi }_{1}+{|{\alpha }_{2}|}^{2}{a}_{1}{\sin }^{2}{\phi }_{2}-x)]\\  &  & \times \,\exp [4Y({|{\alpha }_{1}|}^{2}{b}_{2}{\cos }^{2}{\phi }_{1}+{|{\alpha }_{2}|}^{2}{b}_{1}{\cos }^{2}{\phi }_{2}+y)],\end{array}$$

where $X=-\,\frac{1}{\mathrm{4(}{a}_{1}{a}_{2}-{c}_{1}^{2})},$ $x=8|{\alpha }_{1}||{\alpha }_{2}|{c}_{1}\,\sin \,{\phi }_{1}\,\sin \,{\phi }_{2},$ $Y=-\,\frac{1}{\mathrm{4(}{b}_{1}{b}_{2}-{c}_{1}^{2})}$ and $y=8|{\alpha }_{1}||{\alpha }_{2}|{c}_{2}\,\cos \,{\phi }_{1}\,\cos \,{\phi }_{2}$. This expression can be described as an expectation value of the displaced parity operator. According to Banaszek and Wodkiewicz^33^, photon detection probability for EPR-like state is directly related to the phase space density. Therefore, a Bell type inequality is defined to measure quantum non-locality. The displaced parity operator for a two-mode field becomes $\hat{{\rm{\Pi }}}({\alpha ^{\prime} }_{1},{\alpha ^{\prime} }_{2})={D^{\prime} }_{1}\otimes {D^{\prime} }_{2}$, with ${D^{\prime} }_{i}={D}_{{a}_{i}}({\alpha ^{\prime} }_{i}){(-1)}^{{a}_{i}^{\dagger }{a}_{i}}{D}_{{a}_{i}}^{\dagger }({\alpha ^{\prime} }_{i})$, where ${a}_{i}({a}_{i}^{\dagger })$ is annihilation (creation) operator and ${D}_{{a}_{i}}({\alpha ^{\prime} }_{i})={e}^{({\alpha ^{\prime} }_{j}{a}_{i}^{\dagger }-{\alpha ^{\prime} }_{j}^{\ast }{a}_{i})}$ are the single-mode displacement operators for the two fields. The Wigner function ${W}_{TMGS}({\alpha ^{\prime} }_{1},{\alpha ^{\prime} }_{2})$ in Eq. (13) can be expressed in terms of parity operator as

14 $${W}_{TMGS}({\alpha ^{\prime} }_{1},{\alpha ^{\prime} }_{2})=\frac{4}{{\pi }^{2}}{\rm{\Pi }}({\alpha ^{\prime} }_{1},{\alpha ^{\prime} }_{2}),$$

where ${\rm{\Pi }}({\alpha ^{\prime} }_{1},{\alpha ^{\prime} }_{2})$ is the quantum expectation value of $\hat{{\rm{\Pi }}}({\alpha ^{\prime} }_{1},{\alpha ^{\prime} }_{2})$. Using correlations between parity measurements, the Bell’s combination (${\mathcal B}$) becomes as^32^

15 $$ {\mathcal B} ={\rm{\Pi }}(\mathrm{0,}\,0)+{\rm{\Pi }}(\sqrt{{J}_{1}}\mathrm{,0)}+{\rm{\Pi }}\mathrm{(0,}-\,\sqrt{{J}_{2}})-{\rm{\Pi }}(\sqrt{{J}_{1}},-\,\sqrt{{J}_{2}}),$$

where *J*_1_ and *J*_2_ represents magnitude of the displacements. The Bell’s combination for the TMGS is evaluated as

16 $$ {\mathcal B} =P{ {\mathcal B} }_{1},$$

where $P=\frac{1}{{\pi }^{2}}\int \,\int \,{d}^{2}{\xi }_{1}{d}^{2}{\xi }_{2}\chi ({\xi }_{1},{\xi }_{2})={\mu }_{1}{\mu }_{2}=\frac{1}{4}{[({a}_{1}{a}_{2}-{c}_{1}^{2})({b}_{1}{b}_{2}-{c}_{2}^{2})]}^{-\mathrm{1/2}}$ is the global purity of the output quantum state and

17 $${{\mathscr{B}}}_{1}=1+\exp [\,-\,{J}_{1}{b}_{2}{({b}_{1}{b}_{2}-{c}_{2}^{2})}^{-1}]+\exp [\,-\,{J}_{2}{b}_{1}{({b}_{1}{b}_{2}-{c}_{2}^{2})}^{-1}]-\exp [\,-\,({J}_{1}{b}_{2}+{J}_{2}{b}_{1}+2{c}_{2}\sqrt{{J}_{1}{J}_{2}})\,{({b}_{1}{b}_{2}-{c}_{2}^{2})}^{-1}],$$

with *a*_1_ = *f*_1_ + *f*_11_, *a*_2_ = *f*_2_ + *f*_22_, *b*_1_ = *f*_1_ − *f*_11_, *b*_2_ = *f*_2_ + *f*_22_, *c*_1_ = | *f*_12_| + | *f*_3_|, and *c*_2_ = | *f*_12_| − | *f*_3_|. One can choose *J*_1_*b*_2_ = *J*_2_*b*_1_ or *J*_2_ = *J*_1_*b*_2_/*b*_1_, so ${ {\mathcal B} }_{1}$ can be expressed as

18 $${ {\mathcal B} }_{1}=1+2\exp [-{J}_{1}{b}_{2}{({b}_{1}{b}_{2}-{c}_{2}^{2})}^{-1}]-\exp [-\mathrm{2(}{J}_{1}{b}_{2}+{c}_{2}{J}_{1}\sqrt{{b}_{2}/{b}_{1}}){({b}_{1}{b}_{2}-{c}_{2}^{2})}^{-1}]\mathrm{.}$$

Eq. (16) reveals reduction of the Bell’s combination in value with a decrease in the global purity of the output state. To make ${ {\mathcal B} }_{1}$ maximal, we evaluate *J*_1_ with use of the condition, $d{ {\mathcal B} }_{1}/d{J}_{1}=0$. It results in ${J}_{1}=({b}_{2}+2{c}_{2}\sqrt{{b}_{2}/{b}_{1}})$ ${({b}_{1}{b}_{2}-{c}_{2}^{2})}^{-1}\,\mathrm{ln}(z),$ where $z=(1+\frac{{c}_{2}}{\sqrt{{b}_{1}{b}_{2}}})$. Substituting *J*_1_ in Eq. (18), the optimized Bell’s combination appears as

19 $$ {\mathcal B} =P{ {\mathcal B} }_{1{\rm{\max }}},$$

where ${ {\mathcal B} }_{1{\rm{\max }}}=1+2\,\exp [-{b}_{2}{R}_{1}]\mathrm{ln}(z)-\exp [-\frac{2\,\mathrm{ln}(z)}{{R}_{1}{R}_{2}}]$, with ${R}_{1}={b}_{2}+2{c}_{2}\sqrt{{b}_{2}/{b}_{1}}$ and ${R}_{2}={b}_{2}+{c}_{2}\sqrt{{b}_{2}/{b}_{1}}$. Eq. (19) indicates that the Bell’s combination depends on the parameters ${\mu }_{1},{\mu }_{2},{\tau }_{1},{\tau }_{2}$, beam splitter angle *θ*, phases of the input modes and the phase shift of the beam splitter.

Quantum steering^7,19^ is an interesting quantum mechanical phenomenon which fills the space between entanglement and Bell non-locality on the part of the quantum correlation effect in a system. The steering provides one party with the ability to perform a measurement on their side of an entangled quantum system. As a response, the set of parameters of other party at a non-local distance are influenced. In a composite quantum system with subsystems *A* and *B* associated with the TMGS, *A* steers *B* if the condition ${V}_{AB}+\frac{i}{2}{\mathrm{(0}}_{A}\oplus {{\rm{\Omega }}}_{B})\ge 0$, is not satisfied by Gaussian measurements^19^. Quantum steering is asymmetric in general i.e. ${S}^{A\to B}\ne {S}^{B\to A}$, where steering from *A* to *B* can be quantified with^7^

20 $${S}^{A\to B}=\,{\rm{\max }}\{\mathrm{0,}\,\frac{1}{2}\,{\mathrm{log}}_{2}\frac{{\rm{\det }}\,{\mathbb{A}}}{4\,{\rm{\det }}{V}_{AB}}\}\mathrm{.}$$

The degree of entanglement for bipartite quantum state of a composite system at the output of the beam splitter can be quantified by using logarithmic negativity^11^. The logarithmic negativity can be obtained by ${E}_{n}=\,{\rm{\max }}\,\mathrm{\{0,}-\,{\mathrm{log}}_{2}\mathrm{(2}{\tilde{\lambda }}_{-})\},$ where ${\tilde{\lambda }}_{-}$ is the smallest of the two symplectic eigenvalues under the positive partial transpose^11,51^ of the two-mode covariance matrix *V*_*AB*_, given by $2{\tilde{\lambda }}_{\pm }^{2}={\rm{\Delta }}\pm \sqrt{{{\rm{\Delta }}}^{2}-4\,{\rm{\det }}{V}_{AB}}$ with ${\rm{\Delta }}={\rm{\det }}\,{\mathbb{A}}+{\rm{\det }}\,{\mathbb{B}}$ $-2\,{\rm{\det }}\,{\mathbb{C}}\mathrm{.}$

Quantum discord^40,41^ is another type of quantum correlations which describes the total correlations measurement in a quantum state that is basically the difference between the equivalent expressions of two classical mutual information. We measure the discord within the domain of generalized Gaussian measurements on bipartite system that are described by TMGS of covariance matrix *V*_*AB*_. The general form of Gaussian quantum discord is expressed in Eq. (9).
